# The Great Escape: A Case Series on *DDX3X* Craniofacial Phenotypes in Females

**DOI:** 10.1177/10556656251383776

**Published:** 2025-10-03

**Authors:** Ellen Wang, Kylie Swiekatowski, Danielle Sobol, Matthew Greives, Emily Hansen-Kiss

**Affiliations:** 1 Division of Plastic Surgery, 12339 McGovern Medical School at the University of Texas Health Science Center at Houston and Children's Memorial Hermann Hospital, Houston, TX, USA; 2 Department of Diagnostic and Biomedical Sciences, UTHealth Houston School of Dentistry, Houston, TX, USA

**Keywords:** *DDX3X*, neurodevelopmental disorder, craniofacial, cleft lip and palate

## Abstract

**Introduction:**

*DDX3X*-related neurodevelopmental disorder (*DDX3X*-NDD) is a rare genetic condition that primarily affects females, leading to developmental delays and intellectual disability. *DDX3X* variants, primarily *de novo*, account for an estimated 1% to 3% of females with unknown causes of intellectual disability. Affected males have also been reported, often with the variant being inherited from an asymptomatic or mildly symptomatic mother.

**Case Description:**

*Case 1*. The patient is a 22-month-old female, born full-term, with a history of developmental delay and complete bilateral cleft lip and palate (CLP). Whole exome sequencing (WES) identified a *de novo* pathogenic missense variant in the *DDX3X* gene, *DDX3X* c.1039G > T (p.Asp347Tyr). Developmentally, the patient is progressing gradually—can sit unassisted, but remains nonverbal.

*Case 2*. The patient is a 4-year-old female, born full-term, with a history of right hemifacial microsomia and *DDX3X*-NDD, and no known family history. The patient has global developmental delay, but is able to speak in 3- to 4-word sentences. WES identified a *de novo* *DDX3X* frameshift variant, c.841_842delC>A (p.Gln281AspfsX13). Facial features include right-sided hemifacial microsomia, with right enophthalmos, microtia, mandibular hypoplasia, and bilateral facial nerve weakness. The patient is well-managed at home and receives regular care from neurology and speech therapy at school.

**Discussion:**

Although there have been numerous reports on the neurodevelopmental aspects of *DDX3X*-NDD, craniofacial findings such as hemifacial microsomia and CLP have been minimally reported in the literature. Given the association between *DDX3X* variants and craniofacial findings, plastic surgeons and their multidisciplinary team should be aware of this genetic condition.

## Introduction

*DDX3X*-related neurodevelopmental disorder (*DDX3X*-NDD; OMIM 300958), also known as Snijders Blok type, is a rare genetic condition that primarily affects females, leading to developmental delays and intellectual disability.^1,2^ *DDX3X*-NDD is estimated to occur in 1 in 50 000 live births, and variants account for an estimated 1% to 3% of females with unknown causes of intellectual disability.^3,4^ Affected females present with hypotonia, dysmorphic facial features, behavioral conditions such as autism spectrum disorder and attention-deficit/hyperactivity disorder (ADHD),^5^ various movement disorders, and approximately 50% are nonverbal.^2^ There is wide phenotypic variation within affected females, including reported variants being inherited from asymptomatic or mildly symptomatic mothers.^6^

*DDX3X* maps to Xp11.3-11.23 and encodes a DEAD-box (aspartate-glutamate-alanine-aspartate) RNA helicase, crucial for neural development. DEAD-box helicases are a type of RNA-binding protein that regulate the RNA life cycle through the interaction of ATP and RNA. Females have 2 copies of *DDX3X,* while males have 1 copy of *DDX3X* and 1 copy of *DDX3Y*.^3^ In *DDX3X*-NDD, males have been reported with missense and splice site variants that are generally inherited from an asymptomatic or mildly symptomatic mother or, more rarely, *de novo*.^6,7^ Affected females exhibit a variety of missense, frameshift, and splice site variants,^3,4^ with the majority of reported variants to date being *de novo*.^6^ To date, no truncating variants have been identified in males, suggesting that complete loss of function in the *DDX3X* gene is lethal in males.^7,8^

While males are generally more severely affected in X-linked disorders than females due to X-chromosome inactivation (XCI), *DDX3X* exhibits partial XCI escape.^9^ This phenomenon, in conjunction with skewed X-inactivation, results in the transcription of both wild-type and mutant proteins that act in a dominant-negative manner,^3^ where the normal *DDX3X* protein may not be able to appropriately compensate for the mutant allele. In this report, we describe 2 patients in our Pediatric Plastic Craniofacial Team with pathogenic variants in *DDX3X* presenting with unique craniofacial characteristics that have been minimally reported in the literature.

## Case Presentation

### Patient 1

**Figure 1.**
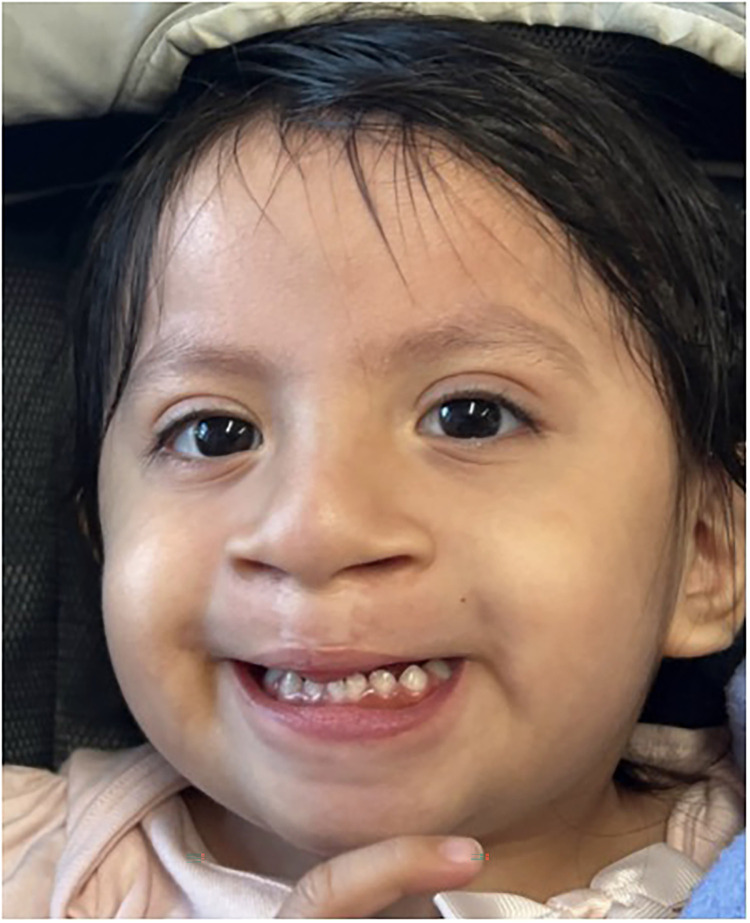
Photograph of Patient 1, a 2-Year-Old Female with Bilateral Cleft Lip and Palate.

The patient is a 22-month-old female (Figure 1 and Table 1), born full-term via vaginal delivery, with a history of global developmental delay and complete bilateral cleft lip and palate (CLP). The patient has no known family history of genetic disorders. Past surgeries include staged cleft lip repair, palatoplasty, and tympanostomy tube placement. Trio whole exome sequencing (WES), a genetic test that compares the protein-coding regions of the patient with those of the parents, identified a *de novo*, heterozygous, pathogenic missense variant in the *DDX3X* gene (*DDX3X* c.1039G > T, p.Asp347Tyr). Developmentally, the patient is progressing gradually—can sit unassisted, crawl, and point to objects, but remains nonverbal. On exam, the patient was noted to have moderate hypotonia, wide nasal bridge, hypertelorism, bilateral epicanthal folds, flat occiput, short neck, and was not weight-bearing. There were no signs of sleep apnea, and the patient follows closely with neurology and speech therapy.

**Table 1.** Comparison of DDX3X-NDD Findings Between the Case Presentations.

*DDX3X*-NDD findings	Case 1	Case 2
Developmental delay	X	X
Intellectual disabilities	X	X
Dysmorphic facial features	X	X
Autism spectrum disorder		X
ADHD		
Aggression		X
Vision/hearing impairment		X
Congenital heart defects		
Scoliosis/joint laxity		
Movement disorders		
Hypotonia	X	X
Sleep apnea		
Epilepsy		

Abbreviations: *DDX3X*-NDD, *DDX3X*-related neurodevelopmental disorder; ADHD, attention-deficit/hyperactivity disorder.

### Patient 2

**Figure 2.**
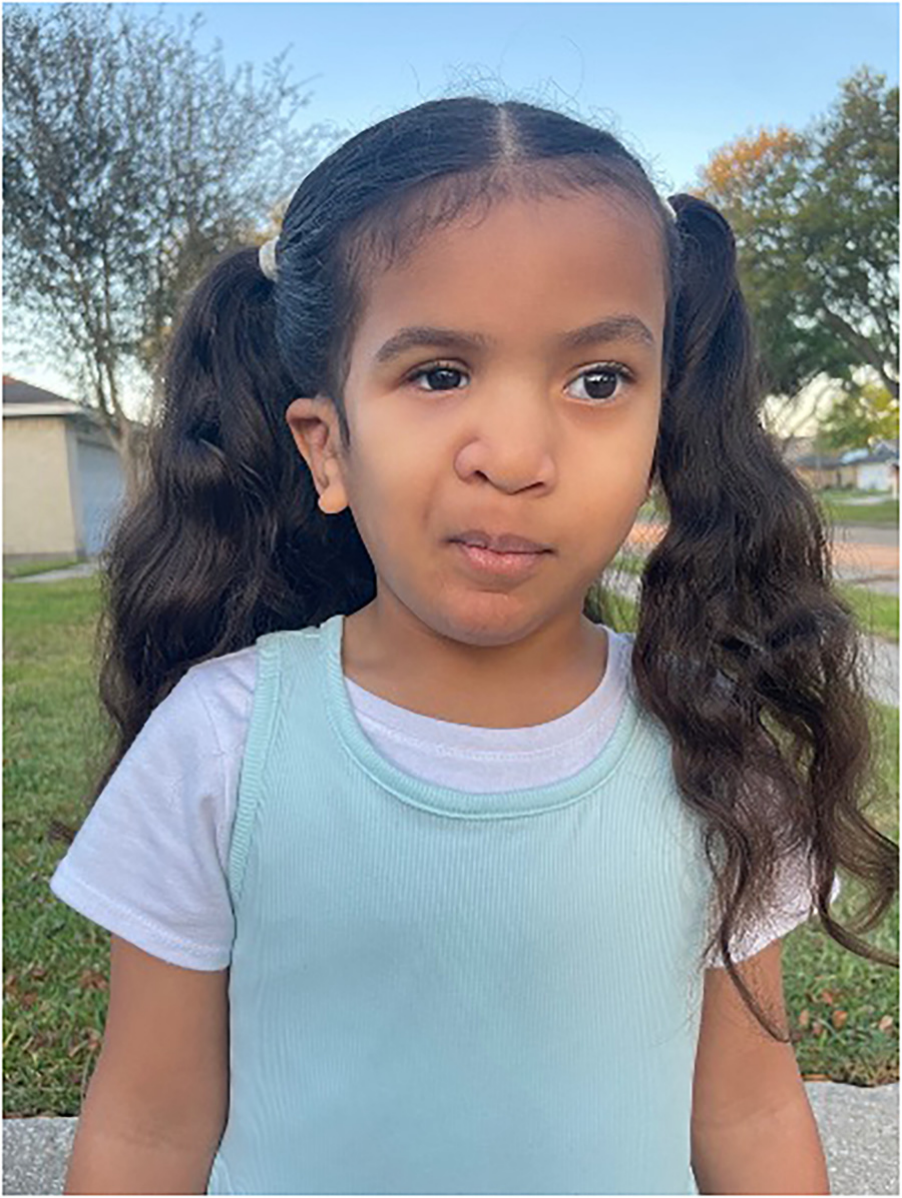
Photograph of Patient 2, a 4-Year-Old Female with Right-Sided Hemifacial Microsomia.

The patient is a 4-year-old female (Figure 2), born full-term via repeat Cesarean section with a brief neonatal intensive care unit admission for mild respiratory distress. The patient has right hemifacial microsomia, global developmental delay, and a more recent diagnosis of autism spectrum disorder. Patient has a history of significant speech delay and is currently able to speak in 3- to 4-word sentences with an estimated 200-word vocabulary. Trio WES identified a *de novo*, heterozygous, pathogenic frameshift variant in the *DDX3X* gene (*DDX3X* c.841_842delC>A, p.Gln281AspfsX13). On exam, the patient was noted to have right-sided hemifacial microsomia with right enophthalmos, blue sclera, right-sided eyelid drooping, bilateral refractive amblyopia, right-sided ear microtia, mandibular hypoplasia, high-arched palate, and bilateral facial nerve weakness. Sleep study and electroencephalogram were both normal. Behaviorally, the patient exhibited some echolalia, stereotypical movements such as humming and hand flapping, hyperactivity, and minor aggression (pinching).

## Discussion

*DDX3X-*NDD is a rare genetic disorder predominantly affecting females that was first identified in 2015 and is associated with intellectual disability (mild to moderate), developmental delays including speech delay, differences in brain structure, hypotonia, ADHD, hearing loss, and heart defects. Additional findings include seizures (16% to 20%), scoliosis (10%), microcephaly (30%), movement disorders, breathing disorders, and precocious puberty.^3^ Facial features include long, hypotonic face, high/broad forehead, wide nasal bridge, bulbous upturned nasal tip, refractive errors, high-arched palate, thin upper lip, large ears, and a smooth and large philtrum.^2,10,11^ Cleft lip with or without palate involvement has been reported in 8% of cases in Snijders Blok et al,^1^ but hemifacial microsomia has not been previously reported. The findings of right enophthalmos, right-sided eyelid drooping, right-sided ear microtia, and mandibular hypoplasia are likely related to the right hemifacial microsomia.

*DDX3X*-related disorder is inherited in an X-linked pattern, where females tend to have a more severe presentation than males. Two mechanisms have been proposed to explain the more severe presentation in females. One explanation is that *de novo* *DDX3X* variations that are sufficiently deleterious to normal protein function are lethal in utero for males.^12^ Importantly, hemizygous deletion of *DDX3X* has been found to be lethal in male mouse embryos, while heterozygous female embryos presented with cardiac defects.^3,8^ On the other hand, overexpression can lead to increased oxidative stress and inflammatory responses.^3^ Both overexpression and underexpression of *DDX3X* can lead to pathological function. It has also been suggested that *DDX3X* exhibits haploinsufficiency in females, and affected males have hypomorphic alleles that retain some normal function; however, there have been some notable exceptions in the literature where *de novo* missense variants have been identified in males.^8^ Another explanation is due to the mechanism of a dominant-negative gene. When 1 copy of *DDX3X* is mutated, it is then unable to be compensated for by the other copy of normal *DDX3X.* In fact, the abnormal protein may actively interfere with normal protein function due to deleterious cellular interactions and competition.^3^ While being an X-linked disorder, *DDX3X* exhibits XCI partial escape where the inactive X chromosome still expresses approximately 30% of the expression of the active X chromosome,^3^ further contributing to the phenotypic heterogeneity of females with *DDX3X*-NDD. Furthermore, different variant types, such as frameshift versus missense, can also result in varying phenotypic severities. Several studies have found that individuals with missense variants were significantly more likely to have more severe phenotypes.^4,12^ However, in our case presentation, Patient 1, who had a missense variant, had a less severe phenotype than Patient 2, who had a frameshift variant, highlighting the wide phenotypic heterogeneity of *DDX3X*-NDD.

Although there have been numerous reports on the neurodevelopmental aspects of *DDX3X*-NDD, craniofacial findings such as CLP and high-arched palate have been reported minimally, and this is the first reported instance of a patient with hemifacial microsomia. Because *DDX3X* plays an important role in Wnt signaling,^1^ a pathway that plays a role in neural crest development,^3^ the disruption to neural crest cells from *DDX3X* variants may affect the development of craniofacial tissue,^13^ especially since hemifacial microsomia is a result of dysfunction in the first and second branchial arches formed by neural crest cells.^14^ Additionally, microcephaly is a common finding, present in one-third of human females with heterozygous germline *DDX3X* variants,^3^ which may also result in dysmorphic craniofacial findings. Hemifacial microsomia presents with unilateral underdevelopment of the jaws and mandible, ear anomalies, and facial asymmetry. In patient 2, the findings of right enophthalmos, right-sided eyelid drooping, right-sided ear microtia, and mandibular hypoplasia are likely related to the right hemifacial microsomia diagnosis. It is possible that the findings associated with hemifacial microsomia in patient 2 are coincidental and unrelated to the syndromic presentation of *DDX3X*-NDD. However, the refractive amblyopia and high-arched palate are consistent with findings that have previously been reported in patients with *DDX3X-*NDD, and it is possible that hemifacial microsomia is a rare finding not previously reported.

This case series contributes to the growing understanding of the phenotypic diversity in females with *DDX3X*-NDD and supports the current literature of reports of CLP and high-arched palate, such as patients 1 and 2, respectively. While it is unclear whether all of the craniofacial findings present in patient 2 are related to *DDX3X*-NDD, craniofacial teams assessing female patients with craniofacial differences and intellectual disability or global developmental delay should include *DDX3X*-NDD on their differential diagnosis.

The clinical presentation of these 2 patients highlights the importance of ongoing craniofacial surveillance in patients with *DDX3X*-NDD and the critical role of genetic counseling and diagnostic testing to facilitate understanding of these diverse genetic conditions for both patient families and clinicians. Given the association between *DDX3X* variations and craniofacial findings, plastic surgeons and their multidisciplinary team should be well-informed of this condition to optimize support for these patients and management of their often complex needs.
